# Systemic Lupus Erythematosus in Pregnancy

**DOI:** 10.3390/medsci13030174

**Published:** 2025-09-04

**Authors:** Angeliki Gerede, Efthymios Oikonomou, Sofoklis Stavros, Anastasios Potiris, Panagiota Papasozomenou, Menelaos Zafrakas, Ekaterini Domali, Nikolaos Nikolettos, Makarios Eleutheriades

**Affiliations:** 1 Department of Obstetrics and Gynecology, Democritus University of Thrace, 69100 Alexandroupolis, Greece; eftoikonomou@outlook.com (E.O.); nnikolet@med.duth.gr (N.N.); 2 Third Department of Obstetrics and Gynecology, University General Hospital “ATTIKON”, Medical School, National and Kapodistrian University of Athens, 12462 Athens, Greece; sfstavrou@med.uoa.gr (S.S.); apotiris@med.uoa.gr (A.P.); 3 School of Health Science, International Hellenic University, 57400 Thessaloniki, Greece; papasozomenou248@gmail.com (P.P.); mzafrakas@gmail.com (M.Z.); 4 First Department of Obstetrics and Gynecology, Alexandra Hospital, Medical School, National and Kapodistrian University of Athens, 11528 Athens, Greece; kdomali@yahoo.fr; 5 Second Department of Obstetrics and Gynecology, University Hospital “Aretaieion”, Medical School, National and Kapodistrian University of Athens, 11528 Athens, Greece; melefth@med.uoa.gr

**Keywords:** systematic lupus erythematosus (SLE), pregnancy, lupus nephritis (LN), adverse pregnancy outcome (APO), preterm birth, neonatal lupus syndrome (NLS), preeclampsia, antiphospholipid antibodies (aPL), complete heart block (CHB), flares

## Abstract

Background/Objectives: The reciprocal relationship between Systemic Lupus Erythematosus (SLE) and pregnancy continues to elude the scientific community’s approaches for a clear understanding. Multiple studies have reached dissimilar results regarding the impact that SLE exerts on pregnancy, whilst the potential risks of lupus pregnancies continue to encumber women of childbearing age. Whether SLE predisposes to a complicated pregnancy and conversely whether pregnancy impacts the progression of the disease is aimed to be assessed by this systematic review. Methods: A thorough search of original research articles was conducted using online databases (PubMed, Google Scholar), initially identifying 877 potential studies. Results: Upon further assessment for relevance and eligibility, 65 articles were selected for detailed analysis. Conclusions: We concluded that, even though advanced approaches have optimized SLE prognosis and treatment, the complexity of the disease requires further extensive study in order to grasp the mechanism behind the susceptibility to adverse complications. SLE pregnancy cannot be considered without risk. Comprehensive, multidisciplinary, and continuous monitoring of the disease course prior to, during, and after pregnancy is necessary to ensure optimal recovery and minimal maternal and fetal complications. Tailored treatments and novel biomarkers would move us towards precise patient-centered care that addresses each patient’s unique disease profile and pregnancy needs, ultimately improving both maternal and fetal outcomes in women with systemic lupus erythematosus.

## 1. Introduction

Systemic lupus erythematosus (SLE) is an autoimmune disease marked by chronic inflammation, a broad spectrum of clinical features, and a complex pathogenesis. The hallmark of the disease is the dysregulation of the immune system, resulting in the production of autoantibodies that target mainly cellular and nuclear components and in inflammation and immune-mediated damage of the tissues through the accumulation of immune complexes [1]. It is characterized by periods of flares and periods of remission.

Lupus exemplifies the complexity of autoimmune diseases with its multifactorial and still not fully understood pathogenesis. Genetic susceptibility, environmental triggers (sunlight, smoking, viruses, e.g., Epstein–Barr), hormonal factors, and regulatory aberrations in the array of immune cells involved lie in the epicenter [2]. Specific alleles for HLA-II, responsible for the antigen presentation, and deficiencies in complement components, particularly C1q, C2, and C4, responsible for immune complex clearance from the tissues, create the backdrop of the disease. Repeated antigen exposure leads to the loss of function of the T-reg cells, the recess of self-tolerance, and the production of antibodies against the now-unrecognized autoantigens. The imbalance in immunomodulation allows the polyclonal B-cell activation and the immune complex deposition in tissues.

These immunological disturbances are further complicated during pregnancy, a state characterized by unique hormonal and immune adaptations that may exacerbate or alter the course of autoimmune diseases, such as lupus. Pregnancy is correlated with changes to the immune modulation, shifts in hormonal levels, and vascular adaptations to support the fetus. Pregnancy induces an unprecedented hormonal and immunological burden. The balance between pro-inflammatory Th1 cell-mediated and anti-inflammatory Th2 humoral cytokines is of the utmost importance in order to ensure tolerance to the semi-allogenic fetus. Generally, a decline in Th1 responses is expected. However, in women with lupus, this modification is not precise enough to avert a major immune dysregulation. The difficulty in fully transitioning to a Th2-dominant state leads inevitably to heightened lupus symptoms and complications with the pregnancy [2].

Fluctuations in hormone levels play a definitive role in the progression of the pregnancy. Profound changes to the hormonal system of the female include elevated levels of estrogens, progesterone, and prolactin. This shift has a significant impact on immune balance. Estrogens, which increase during pregnancy, extend the survival of B lymphocytes, as well as promote their proliferation, differentiation, and antibody-producing ability, through the production of IL-10. Enhanced antibody production ability justifies the higher frequency of SLE flares during pregnancy. Given the deleterious effects of this exposure [3,4], an overall negative influence is expected. Progesterone, alongside the human chorionic gonadotropin (hCG), typically downregulates the immune function, whilst the way they modulate the activity of SLE remains unknown. Although heightened, their protective role against pro-inflammatory effects may be insufficient. Extrapituitary prolactin (PRL) and interleukins, mainly IL-6, produced by lymphocytes from patients with active SLE, create a positive feedback loop and stimulate the overexcretion of prolactin, as well as create defects in the dopamine metabolism and peptidergic modulators. Prolactin also acts as an up-modulator of B-cell survival and humoral immunity, exacerbating the SLE effects during pregnancy. Little doubt is left as to whether pregnancy and SLE activity are intertwined.

Chronic inflammation caused by Lupus, combined with the deposition of immune complexes, is associated with endothelial dysfunction. This dysfunction, combined with the changes to the vascular system and to the placenta, in order to facilitate the increased blood flow and the space for the developing fetus, may prove fatal, due to the increased possibility of thrombosis, intrauterine growth restriction, and lower capacity for amniotic fluid.

Lupus is known to imitate the symptoms of a variety of other diseases, deterring clinicians from reaching a definitive diagnosis and the appropriate treatment. Chronic undiagnosed problems of the skin, bones, lungs, eyes, as well as complications involving the renal and hematological system, are usually the predecessors of SLE. The outbreak of lupus includes manifestations in any of the above-mentioned systems. Hypertension, renal dysfunction (Lupus Nephritis), low bone mass, and skin lesions are the most prevalent ones.

SLE presents a strong female predilection and affects more commonly women of childbearing age [5]. The disproportionality of lupus prevalence, with a female-to-male ratio reaching 9:1, depends on various genetic, hormonal, and socioeconomic factors. The mean age of disease appearance in women is around 30 [6]. Development of the disease can also be attributed partly to pregnancy, as it is not rare for previously healthy women to develop lupus-like symptoms postpartum. The change in cytokine profile and the hormonal shift, as discussed later in the review, are the identified culprits.

Conventionally, the medical community advised against pregnancy in SLE patients, since the fetal and maternal well-being of such patients was in grave peril. Advancements in medicine nowadays facilitate childbearing in women prior to or even after lupus onset, posing a great challenge to the medical specialists. The findings on pregnancy and lupus are variable and in many cases contradicting; the recommendations with regard to management of the disease differ [7]. The objective of this systematic review is to provide a comprehensive summary of the available data, explore the correlation between pregnancy and SLE, assess risk factors for poor outcomes, discuss management strategies, and address the gap between scientific findings.

## 2. Materials and Methods

Study design: Our methodology utilized a structured and comprehensive approach to assess how SLE influences pregnancy outcomes, as well as how pregnancy impacts SLE disease activity. An extensive literature review was carried out to identify pertinent studies, which were then screened using clearly defined inclusion and exclusion criteria.

**Figure 1:**
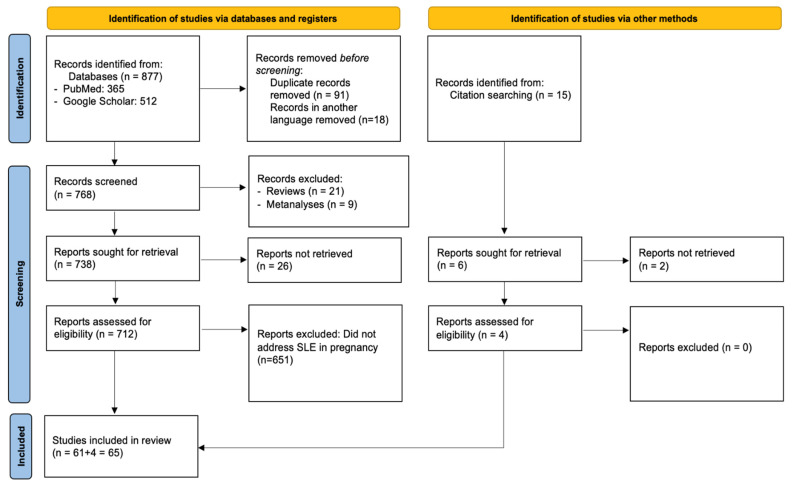
Flow diagram of the selection process of the included studies according to PRISMA 2020 guidelines.

PRISMA compliance: This systematic review was conducted and reported in accordance with the PRISMA 2020 (Preferred Reporting Items for Systematic Reviews and Meta-Analyses) guidelines to ensure methodological transparency and reproducibility. A structured protocol was developed prior to the literature search, specifying the objectives, eligibility criteria, data extraction process, and quality assessment strategy. Although the protocol was not registered in any database, the full methodology is detailed within this manuscript. A PRISMA flow diagram summarizing the study selection process is presented in Figure 1, and a complete PRISMA 2020 checklist is provided in Supplementary Materials.

Literature search: Relevant studies were identified by searching PubMed and Google Scholar. Keywords related to SLE, pregnancy, and safety were applied, using Boolean operators, such as “AND” and “OR”, to refine the search strategy, i.e., << “Systemic Lupus Erythematosus” OR “SLE” AND “pregnancy” OR “gestation” AND “Safety” >>. The search algorithm maintained a common structure across databases; however, adjustments were made to ensure optimal search results. Additionally, we proceeded to hand-search the reference lists, reviews, and grey literature for relevant articles that the search methodology did not identify.

Studies selection and eligibility: This review included a range of study types—observational, retrospective, and prospective cohort studies, as well as case–control studies—that examined the impact of SLE on pregnancy progression, maternal health, and fetal outcomes. Literature published up to December 2024 was considered. Studies were included if they involved women with SLE during pregnancy, the peripartum or postpartum period, or if they addressed clinical aspects of reproductive health relevant to women of childbearing age with SLE. This included studies on pregnancy planning, fertility, contraception, and postpartum disease activity, provided that the population remained consistent with the review objectives. We excluded studies that did not address pregnancy, reproductive-related issues, or studies of drug-induced lupus. Some clinical studies focused on specific clinical subtypes of SLE, such as lupus nephritis or childhood-onset SLE. These were included only if the primary disease was classified as SLE, and their outcomes were analyzed in the context of pregnancy. In a limited number of cases, studies not directly reporting pregnancy outcomes were retained, because they addressed clinically relevant aspects, such as diagnostic consistency [8] or management of pregnancy-associated complications [9]. Reviews, meta-analyses, animal studies, and articles not published in English were also excluded.

Following the initial search, two independent reviewers screened titles and abstracts to determine relevance. Any conflicts were settled either by mutual agreement or with the involvement of a third reviewer. Irrelevant records were removed. Rayyan was used to perform semi-automated deduplication of the results. The remaining full-text articles were then evaluated for eligibility based on PICOS criteria by two blinded reviewers. Discrepancies were again resolved via consensus or input from a third reviewer. Additionally, reference lists of the included full texts were examined to capture any potentially overlooked studies using a snowballing approach. No eligible articles were excluded due to reviewer disagreement.

Data extraction: Both topic-specific data and general study information were collected from each included article. The extracted information from eligible studies included the following elements: Year of publication, country, study design, study population, and APOs were defined as one or more of the following: miscarriage, stillbirth, intrauterine growth restriction (IUGR), preterm delivery, preeclampsia, neonatal death, or low birth weight, as defined by the individual studies. Information on immunological markers, such as antiphospholipid antibodies (aPL), when reported, including lupus anticoagulant, anticardiolipin antibodies, and anti-β2 glycoprotein antibodies, was also extracted.

The extracted data were organized into a tabular format, with all entries managed and stored using an Excel spreadsheet. In instances where data were missing or unclear in the original publications (e.g., number of pregnancies or specific outcome definitions), we recorded these fields as “Not Applicable, N/A”. No assumptions were made to impute missing data. The authors were not contacted for additional information due to the high volume of included studies and resource limitations. Where relevant, we highlighted gaps in data availability in our tables and discussion, especially where missing information might affect the interpretation of findings.

Quality Assessment: To evaluate the methodological quality and risk of bias of the included observational, case–control, and cohort studies, we employed the Newcastle–Ottawa Scale (NOS). This tool is specifically designed for non-randomized studies and evaluates quality based on three broad domains: selection of study groups, comparability of groups, and ascertainment of the exposure or outcome. Each study was independently assessed by two reviewers, with any disagreements resolved through discussion with a third reviewer. The NOS score ranges from 0 to 9. Studies scoring 8–9 points were classified as low risk of bias, those scoring 6–7 points as moderate risk, and those scoring ≤5 points (none in our dataset) would be considered high risk of bias.

## 3. Results

### 3.1. The Selection Process of Included Studies

Figure 1 illustrates the study selection process, along with reasons for exclusion. The initial database search retrieved 877 articles, 365 from PubMed, and 512 from Google Scholar. After removing 91 duplicates and 18 non-English papers, 768 records remained for title and abstract screening. During this phase, 637 were excluded for not focusing on SLE in pregnancy, 21 were review articles, and 9 were meta-analyses. This left 61 studies for full-text review, all of which fulfilled the inclusion criteria and were incorporated into the final analysis. No full-text articles were excluded at the eligibility stage. As such, a table of excluded studies with appropriate justifications, as recommended by PRISMA 2020 (Item 16b), is not applicable in this case. This decision reflects the strict application of our inclusion criteria during the abstract/title screening phase, which resulted in high eligibility among full texts.

Among the included studies, 32 were obtained via Google Scholar and 29 from PubMed. In addition, reference lists of these articles and citations from other pertinent publications in high-impact journals were hand-searched, and 4 papers that were lost from the initial literature search were included as well. Thus, the 65 studies that were included in total investigated in depth the association between SLE and pregnancy. Across the 65 included studies, a total of 7808 pregnancies in women with SLE were reported. Eight studies did not specify the exact number of pregnancies and were therefore not included in this total. The comparative study by Clowse et al. (2008), investigating SLE in 16.7 million pregnancies, was also not included in the total [10]. The characteristics of the included studies can be found in Table 1.

A formal quality assessment using the Newcastle–Ottawa Scale was performed for all 65 included studies. The majority were found to have a moderate to low risk of bias. Specifically, 28 studies (42%) were rated as low risk, 37 studies (58%) were rated as moderate risk, and no studies were rated as high risk. Common limitations included insufficient adjustment for confounding factors, missing data on follow-up, and non-representative sampling in retrospective designs. Nevertheless, the overall quality of evidence was deemed sufficient to support the review’s conclusions. A full summary of NOS scoring is provided in Table 2.

A considerable degree of heterogeneity was observed across the included studies. This heterogeneity arose primarily from variations in study designs (retrospective vs. prospective cohorts, observational studies, case–control designs), geographic diversity of patient populations, and inconsistency in outcome definitions. For instance, studies used different thresholds and biomarkers to define disease flares, preeclampsia, or APOs. Furthermore, some studies focused specifically on lupus nephritis or antiphospholipid syndrome, while others assessed general SLE populations, which may influence both maternal and fetal outcome patterns.

Differences in healthcare systems, treatment access, and follow-up duration also contributed to the variability in reported findings. Some datasets relied on self-reporting or limited diagnostic criteria, which may under- or overestimate associations. Although the systematic methodology and quality assessment help mitigate these limitations, such heterogeneity restricts the potential for direct comparisons and the formulation of uniform conclusions. Therefore, the findings of this review should be interpreted with an understanding of this underlying variability.

Thirty-seven of the included studies reported information on lupus flares during pregnancy. However, the definitions and methods used to identify flares varied considerably between studies. Among the studies that reported flare data, the majority observed flares in 15–40% of SLE pregnancies. In studies specifying severity, most flares were described as mild to moderate and managed with corticosteroids or adjustment of baseline immunosuppression. Severe flares requiring hospitalization were uncommon. The most frequently reported organ systems involved included the renal system (lupus nephritis), followed by hematologic, dermatologic, and musculoskeletal systems. Due to heterogeneity in definitions and reporting, the exact number of flares could not be aggregated. Nonetheless, flare occurrence was generally lower in patients with quiescent disease at conception. Notably, flare rates in non-pregnant SLE populations over comparable timeframes have been reported to range from 25% to 35%, depending on baseline disease activity and treatment adherence. The flare frequency observed in pregnant women in this review appeared similar or slightly lower, likely reflecting close monitoring and careful disease planning prior to conception.

Fifteen of the included studies specifically investigated predictors of APOs in women with SLE. These studies collectively included data from approximately 2093 pregnancies. The most commonly evaluated predictors included disease activity at conception or during pregnancy, antiphospholipid antibody (aPL) positivity, lupus nephritis, previous APOs, and use of immunosuppressive medications, such as corticosteroids or hydroxychloroquine. The majority of studies identified a clear association between active disease (particularly lupus nephritis or serologic activity) and increased risk of preterm birth, miscarriage, or other APOs. aPL positivity, especially lupus anticoagulant, was consistently linked to a higher risk of fetal loss, preeclampsia, and IUGR. Conversely, the use of hydroxychloroquine appeared to be protective.

### 3.2. Effects of Pregnancy on Lupus

The majority of the literature suggests that pregnancy is associated with an elevated risk of lupus flares. This increase fluctuates depending on the study sample from 25 to 65% [5,28]. Lupus flares are, in most cases, mild and affect mainly the musculoskeletal, hematological system, and the skin [73]; therefore, they are easily confused with the natural changes caused by pregnancy itself, hindering the categorization of the two. A reference should be made to the fact that de novo flares of SLE are seldom in pregnancy, accounting for less than 2% of the cases [65,66].

Pregnancy environment can either exacerbate [74,75,76] or mitigate lupus activity [8,42]. Some women report disease quiescence; however, the majority experience a significant increase in the frequency of flares. The effects of lupus may even remain unchanged. The effect appears highly individualized and dependent on lupus activity at the time of conception [8,42,75,76], the tissues involved, and the type of aberrant antibodies produced. There are even documented cases of women who experience flares in between pregnancies [22,76] and women with lupus onset only after pregnancy [32]. It is also plausible that exposure to pregnancy in SLE patients may cause long-term effects on the course of the disease [13]. Severe mood changes, depression, increased disease activity, and low bone density are common, particularly within three months post-partum [28].

Lupus activity levels at the time of conception and prior nephrological problems, due to SLE, are major predictors of potential flares. Therefore, it is a generally established requirement in pregnancy risk stratification to schedule childbearing at least 6 months later than an active flare. Zhang et al. [69] supported this clinical framework for preconception optimization based on a large Chinese cohort. The study recommends that women achieve low disease activity, have no active organ involvement, and maintain stable treatment for at least six months before attempting conception. This approach was also supported by Zucchi et al. [72]

In combination with the absence of renal disease, these are auspicious markers for a healthy pregnancy progression. Notably, the renal histological pattern obtained years before pregnancy is not predictive of pregnancy progression [35].

The proportion of pregnancies leading to live births is notably lower after the onset of the disease. Likewise, miscarriages, preterm births, and fetal losses are remarkably more prevalent after the onset in comparison to prior, as Al Arfaj et al. concluded in their retrospective study [12].

### 3.3. Effects of Pregnancy on Lupus

The impact of SLE on pregnancy is significantly more understood [26]; however, it remains a matter of debate whether Lupus negatively impacts the progression of the pregnancy.

Prior to pregnancy, the manifestations in women are mostly cutaneous and articular [75]. Women with SLE generally demonstrate fertility rates analogous to unaffected individuals and similar to the general population [18]. Predisposition to lower fertility can be induced due to factors, like renal complications and use of medications, such as high doses of steroids and treatment with alkylating agents, e.g., IV cyclophosphamide [65,77]. To minimize gonadotoxicity, due to this class of medications, an adjuvant treatment with a GhRH agonistic analog may be considered.

APOs are becoming progressively less common, possibly due to better risk stratification and management, as well as treatment improvements. APOs include the following:

- Preterm birth: Preterm birth is considered the most common adverse outcome in SLE pregnancies, as it occurs in over a third of the cases [10,65]. Elevated lupus activity, even if only serological without clinical manifestations, inappropriate treatment with steroids (high prednisone use causes rupture of the placental membranes and acute fetal distress [18]), high blood pressure, thyroid disease [53], and severe renal failure might cause irregularities to the menstrual cycle, amenorrhea, and are the main contributors to a preterm birth [78].
  Given the fact that a successful pregnancy outcome is correlated with a gestational age of at least 32 weeks [27,53], the prevalence of preterm birth needs to raise serious concerns to the scientific community with regard to the management of the disease.
- Preeclampsia: Preeclampsia is characterized by the pregnancy-specific induction of hypertension and proteinuria, which lead to significant end-organ damage, pregnancy losses, and IUGR, among others. Preeclampsia’s frequency is reported doubled to tripled in lupus pregnancies in comparison to healthy ones [19]. Preeclampsia resembles lupus nephritis (LN), presenting as a conundrum to differential diagnosis. Lupus nephritis is an inflammation of the kidneys and a result of SLE. Preeclampsia can also occur in healthy pregnancies and does not pose a threat of similar range. The distinction between the two lies upon the decreasing complement levels, increasing dsDNA antibody levels, and the urine sediment in LN. The prompt diagnosis is also critical, as a different approach is adopted in each case: delivery for preeclampsia (often depicted in study results as cesarean section) and immunosuppression for SLE [10]. We cannot rule out that the studies exploring LN/preeclampsia, which were included in our systematic review, were not biased against the differentiation between them; however, it is notable that at least a fifth of pregnancies are complicated by hypertension and/or proteinuria. In a comprehensive regional analysis, Cajamarca-Baron et al. [68] reported high rates of adverse outcomes, including preeclampsia (up to 52%) and preterm birth (up to 70%). The presence of lupus nephritis nearly doubled the risk of preeclampsia (RR = 1.89), underscoring the need for aggressive renal disease monitoring and tailored care in this population.
  Preeclampsia and lupus nephritis are more common in women with impaired renal function; however, deterioration of renal activity need not discourage pregnancy, even though the risk of premature birth and preeclampsia is elevated [33,59,61].
- Antiphospholipid antibodies (aPL): Antiphospholipid antibodies, i.e., anticardiolipin and lupus anticoagulant, are present in 25–50% of lupus pregnancies. The complications are attributed to thrombosis in the uterine vasculature, due to the inactivation of coagulation factors, as well as to the binding of antibodies to trophoblasts, endothelial, and neuronal cells. aPL antibodies are closely associated with IUGR and fetal morbidity, as a twofold increase in fetal loss is documented in comparison to aPL-negative pregnancies [12,26,50]. Notably, a few studies found no association between aPL antibodies and fetal loss [49].
  It is necessary to distinguish the presence of aPL antibodies from primary antiphospholipid syndrome (APS). aPL antibodies may occur without coexisting lupus, posing a risk to pregnancy. Factually, aPL presence without APS shows an elevated risk of APOs, like intrauterine growth retardation and preterm births [64].

Overall, APOs were reported in a large proportion of included studies. Preterm birth was the most frequently described complication, reported in 34 studies, followed by miscarriage (29 studies), intrauterine growth restriction (17 studies), stillbirth (11 studies), low birth weight (10 studies), and neonatal death (7s studies). The definitions of these outcomes varied between studies, limiting direct aggregation. However, preterm delivery and miscarriage were consistently the most commonly reported APOs. Several studies also stratified outcomes by aPL status. In these studies, the presence of aPL was associated with increased rates of miscarriage, stillbirth, and preeclampsia, consistent with the known prothrombotic effects of these antibodies. Additionally, a higher frequency of APOs was observed in studies involving patients with active lupus during pregnancy or those receiving suboptimal immunosuppressive treatment. The frequencies of the reported APOs across the 65 included studies are depicted in Table 3.

The frequency of APOs in SLE vs. in non-SLE patients is described in the study by Clowse et al. [10], where the data clearly illustrate a significantly higher incidence of APOs in women with SLE compared to those without, based on a large sample of 16.7 million pregnancies over four years in the US. For instance, Cesarean sections were notably more common in SLE deliveries (36.6%) than in non-SLE deliveries (25.0%). Similarly, preterm labor affected over a fifth of SLE pregnancies (20.8%) compared to just 8.1% of non-SLE pregnancies, translating to 2.4 times higher odds for SLE patients. IUGR also showed a substantial difference, occurring in 5.6% of SLE deliveries versus 1.5% of non-SLE deliveries. The most striking disparities were observed for hypertensive disorders of pregnancy: Preeclampsia was nearly three times more frequent in SLE pregnancies (22.5%) than in non-SLE pregnancies (7.6%), while eclampsia, although rare overall, was 4.4 times more likely in SLE deliveries (0.5%) than in non-SLE deliveries (0.09%). Across all listed complications, the statistically significant *p*-values (<0.001) consistently affirm that these adverse outcomes are substantially more prevalent in pregnancies affected by SLE [10]. Clowse et al. underscored that when adjusted for the increased maternal age of the women with SLE, the risks for preeclampsia, preterm labor, and IUGR remain unchanged [10]. This gives us a serious insight into the mechanism of the disease and proposes potential unexplored aspects of management.

The parallel presence of preeclampsia and APS may lead to the most serious form of preeclampsia: hemolysis with elevated liver tests and thrombocytopenia (HELLP) [21]. A most accurate name for this progression would be “complementopathy”, as a genetic defect in complement proteins or an autoantibody against complement components is responsible. Pathological immunomodulation and immunosuppressive therapy also pose an increased risk for infection, mainly pneumonia and sepsis [10]. Gestational diabetes mellitus has also been reported to be present during SLE pregnancies, even though additional studies are deemed necessary for conclusions to be drawn.

### 3.4. Pregnancy Complications

Besides preterm birth, fetal complications include intrauterine fetal loss, perinatal mortality, low birth weight, delayed development for gestational age, and admission to the neonatal intensive care unit (NICU) [11,18,24].

The presence of antibodies plays a definitive role in the progression of fetal health during and even after pregnancy. During pregnancy, aPL antibodies lead to a hypercoagulate state and possibly to intrauterine growth restriction and fetal death [12,26]. Postpartum, the newborn may be affected by the onset of neonatal lupus erythematosus.

Neonatal Lupus Erythematosus—Neonatal Lupus Syndrome (NLS): A form of temporary passively acquired fetal autoimmunity from maternal antibodies that cross the placenta. Neonatal lupus is usually benign and self-limited, as it lasts approximately 6 to 8 months after birth (the time that maternal antibodies remain in the circulation of the neonate) [5]. Neonatal LE is strongly associated with the presence of antibodies against the cytoplasmic ribonucleoproteins: anti-Ro/SSA and anti-La/SSB antibodies. Their coexistence is reported in 10% of the cases [18,36].

Manifestations of NLS include a red raised rash (subacute lupus lesions), along with hematologic and hepatic abnormalities (enlargement of the liver and the spleen). The above seem to resolve after clearance of the maternal antibodies. However, injury to the developing fetal cardiac conduction pathway and cardiac structural abnormalities may lead to permanent damage, also known as congenital heart block [18,58]. It can develop as early as the fourth month of gestation or even in the postpartum period. Its most serious complication is Complete Heart Block (CHB). CHB is preceded by various degrees of conduction delays.

Two percent of neonates exposed to SSA or SSB antibodies are at risk of CHB. Prompt diagnosis can delay but not reverse its progression, leading to fetal mortality in a fifth of the cases. In the rest of the cases, a pacemaker is the only viable solution. Given the pathophysiology of CHB, it presents a relatively high recurrence rate, which reaches 20% [49]. Cardiac monitoring for fetuses and neonates is imperative.

Maternal anti-nuclear antibodies (ANAs)’ association with adverse fetal complications has not been elucidated yet. The presence of anti-nuclear antibodies (ANAs), based on today’s scientific research, could be considered only a predictor and nothing more [39].

### 3.5. Predictors of Pregnancy Outcomes

New ways of studying lupus, as well as the number of studies published on SLE, have contributed to the definition of predictors of pregnancy outcomes, favorable or not. Lupus nephritis, preeclampsia, C3, C4 complement components, hypertension, anti-Ro, anti-La, aPL antibodies, SLE flares, age, disease activity, duration, and IV cyclo-therapy were evaluated as potential predictors.

Lupus nephritis is considered almost unanimously a determinant of unfavorable prognosis [33]. Lupus nephritis contributes significantly to renal and overall damage accrual. The results of a few studies, however, are not definitive regarding the robustness of LN as an APO predictor [12]. Preeclampsia did not present such a correlation. High possibility of organ damage is commonly associated with older age [10], high doses of glucocorticoid medication, history of chronic hypertension [50], disease activity, and duration before and at conception. Hypertension and the use of steroids alone do not fulfill the criteria of a prognostic factor [19]. Lupus quiescence presents strongly as such [7,29]. The traditional approach that conception should take place at least 6 months after a flare has been shown to reduce the possibility of a pregnancy flare and promote the healthy progression of the disease [13,17].

The more elevated the presence of autoantibodies, the more intense the fetal complications. Secondary antiphospholipid syndrome, anti-Ro, and anti-LA antibodies are strongly associated with spontaneous abortions and prematurity [12,26,37,52,53]. Anti-Ro antibodies are a marker of an unfavorable prognosis in both SLE and non-SLE pregnancies; hence, they cannot be utilized as predictors for SLE pregnancy outcomes. Antinuclear antibodies are not predictive, as despite having a negative ANA titer, women can experience significant complications [7]. Cases, which combine either high SLE activity and hypocomplementemia or high anti-dsDNA antibodies titer in the second trimester, are associated with the highest rate of pregnancy loss and preterm birth [23,25]. Hypertension was concluded as a predictor, yet weak, for intrauterine growth restriction [12]. Buyon et al. noted the low C3 and C4 at baseline were also associated with high risk of de novo flares [17], but this is only a standalone result.

Positive reaction to low dose aspirin therapy, which is used for its protective role against SLE, is an unbased, yet confirmed favorable predictor [27,35]. Likewise, proteinuria is an easy, immediate way to assess the possibility of flare presentation, but not established [35].

Overall, the risk of bias cannot be excluded from the assessment of the results; hence, given the dissimilarity of findings, only Lupus Nephritis, aPL antibodies, age, activity of disease at conception, and SLE flares during pregnancy can be employed as solid, reliable predictors. Glycocorticoids, although no study rejected their correlation with an unfavorable outcome, lack sufficient evidence to prove their reliability.

### 3.6. Disease Management

Different approaches regarding the management of the disease are adopted depending on the different pregnancy timepoints. Prior to conception, it is advisable for women with SLE to discuss their intentions with professionals, so as to assess the relevant risks. Pregnancies in individuals with SLE should be planned; hence, contraception is of the utmost importance in these cases. Women ought to be advised against pregnancy in the case that a comorbidity strongly associated with lupus is already present. These include renal deterioration or failure, hypertension, lung disease, heart problems, stroke, and prior pregnancies with severe preeclampsia or hemolysis, along with elevated liver enzymes and low platelets (HELLPs) [14]. To avoid possible complications, pregnancy should be planned for a period of minimal disease activity, as discussed previously in this review. To ensure that, in addition to routine tests, extensive serological tests are required to define lupus activity levels: urine analysis, titer of complement components, and aPL antibodies, among others. When a young woman experiences severe LN complications, an aggressive therapy, including cytotoxic agents, is proposed to ensure minimum levels of proteinuria at the conception time point [35].

The use of antimalarials, such as hydroxychloroquine (HCQ), can be effective in limiting SLE flares, without posing a threat to the fetus [28]; however, inactive instead of controlled disease during the conception period is reported to be a favorable factor against preterm birth and immaturity [22]. HCQ can be used both prior to and after conception, and overall, it is the most trusted medication against SLE, which has been used historically [28,67,71]. It is prescribed to at least half the women with SLE during pregnancy and to 85% of women in the post-partum period [28]. Immunosuppressive drugs, such as azathioprine, and steroids, such as prednisone, at the lowest possible dosage schemes do not have to be stopped before conception and can be continued during pregnancy. Methotrexate and immunosuppressants, like mycophenolate and cyclophosphamide, are teratogenic; therefore, it is mandatory to stop them before conception. Currently, there is limited research regarding the use of biologics, like belimumab and rituximab, during pregnancy [79]. As of today, their discontinuation is advised. Supplementation with folic acid and vitamin D is associated with better lupus management and is recommended generally during pregnancy.

Successful pregnancy outcomes are strongly correlated with a gestational age of more than 8 months, making careful monitoring and therapeutic management necessary [27,53]. An immense challenge for clinicians is the overlap of lupus features with the complications of pregnancy itself. Antimalarials should be continued throughout pregnancy if they were necessary for controlling the disease prior to conception. Low-dose aspirin therapy can be used throughout pregnancy for its protective role against organ damage, a role similar to HCQ [27,28,35]. Non-steroid anti-inflammatory drugs should be stopped around the 8th month, as they hinder the completion of fetal organ formation and lead to defects. Fluorinated steroids (dexamethasone, betamethasone, etc.) can be used only once and only in critical situations. Fluorinated steroids, IVIg, and β-agonists are usually used in the case of heart block. To manage high titers of antiphospholipid antibodies, the use of antithrombotic drugs, mainly antiplatelets, e.g., heparin, is the protocol. Similarly, appropriate therapy needs to be instituted for hypertensive diseases.

Medications used for controlling lupus (except biologics for which insufficient data are collected) are considered safe during breastfeeding, as they are transferred into breast milk in minimal amounts. Neonatal lupus is self-resolving, and usually conservative treatment is sufficient until the clearance of the maternal autoantibodies.

**Figure 2:**
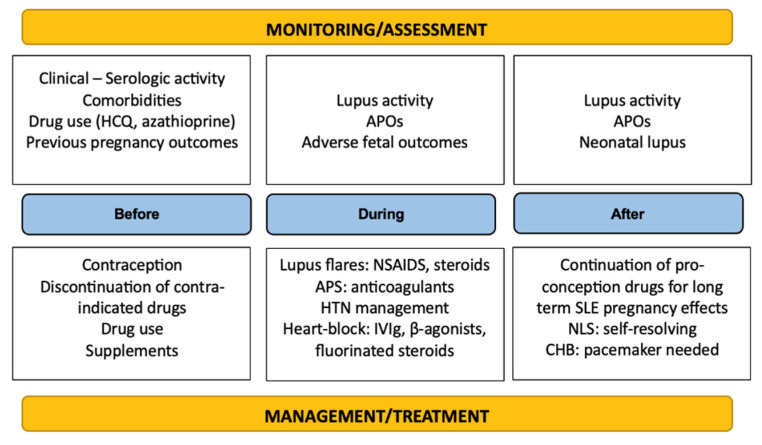
Summary of monitoring and management principles in SLE.

It is easily deducible that multidisciplinary and continuous (rheumatological, hematological, cardiological, and obstetrical) management and close monitoring of SLE pregnancies progression are necessary throughout the gestation period to increase the chances of a successful pregnancy and even after birth to ensure optimal recovery and minimal complications. A summary of the therapeutic management and surveillance of the disease is depicted in Figure 2.

## 4. Discussion

SLE in pregnancy creates a complex interplay of immunological and physiological challenges, a cascade of inflammatory responses often resulting in serious maternal and fetal complications. In this systematic review, a variety of articles were assessed, in order to obtain a better understanding of the latest findings. Contradictory studies, dissimilar and mutually exclusive results, perplex the scientific community. Considerably variable rates in published reports, most of which are retrospective analyses, regionally restricted studies, and overall differences in study populations, the number of patients included, in the methodology of the study, and the existence or lack of a control group, play a definitive role.

The nature of the disease itself makes it difficult for scientists to further study it. The inconsistency of flare definition hinders researchers from making comparisons across studies. The similarity of manifestations with other diseases or even with the natural pregnancy state does not exclude the risk of bias regarding the inappropriate categorization.

The heterogeneity among the studies included in this review represents both a challenge and a strength. On the one hand, the diversity in study types, geographic regions, and clinical focus areas (e.g., lupus nephritis, anti-Ro positivity, or aPL syndrome) complicates efforts to draw universally applicable conclusions. On the other hand, it offers a comprehensive reflection of the real-world complexity and the global nature of managing SLE in pregnancy. This variation underlines the necessity for individualized, patient-centered approaches rather than generalized guidelines. It also emphasizes the need for large, prospective, multicenter studies with standardized definitions and outcomes to improve future comparability and strengthen evidence-based recommendations.

Despite the systematic and methodologically rigorous approach employed in this review, several limitations should be acknowledged. First, publication bias may have influenced the findings, since studies reporting negative or non-significant outcomes are often underrepresented or harder to locate in conventional databases. Additionally, language bias was introduced by limiting included studies to those published in English. Second, a substantial challenge lies in the differentiation between SLE flares and physiological symptoms of pregnancy, such as fatigue, joint pain, or mild edema, which are common to both conditions. This overlap may lead to misclassification bias and complicate flare reporting and assessment. Third, variations in follow-up periods, definitions of adverse outcomes, and confounder control limited our ability to synthesize findings more quantitatively. Some included studies also had incomplete reporting of key variables, leading potentially to limited completeness of synthesis for certain outcomes. Finally, the lack of randomized controlled trials (RCTs) in this field restricts the evidence to largely observational designs, which are inherently more prone to bias.

This review underscores the need for harmonized definitions and diagnostic criteria for pregnancy outcomes in SLE patients. Future studies should aim for prospective, multicenter designs that include diverse ethnic populations to increase generalizability. The long-term postpartum disease trajectory in women with SLE and strategies to differentiate physiological pregnancy changes from early lupus flares should definitely stand at the epicenter of these studies. Additionally, the role of biomarkers (e.g., anti-Ro, anti-dsDNA, and complement levels) in predicting pregnancy outcomes, as well as the safety and efficacy of newer biologics in pregnancy, including belimumab and rituximab, should be further assessed. Finally, the impact of social determinants of health and access to care on SLE pregnancy outcomes should not be overlooked, as it may provide significant insights into the heterogeneity of study results. In clinical practice, this evidence supports a need for personalized management plans, early risk stratification, and multidisciplinary monitoring tailored to each patient’s immunologic profile and obstetric history.

Pregnancy in women with SLE is not without risk. While advances in therapeutic approaches and risk stratification have remarkably contributed to improved prognosis, the lack of predictability of the disease course and susceptibility to complications highlight the need for individualized, multidisciplinary treatment. Biologics seem to be the protagonists in a new era of lupus treatment. There is no doubt that large-scale, multicenter studies are crucial in defining the exact mechanism of the disease and identifying novel biomarkers and tools, so as to ultimately foster safer pregnancies for SLE patients.

## 5. Conclusions

SLE during pregnancy remains a high-stakes clinical scenario with significant implications for both maternal and fetal outcomes. While prognosis has markedly improved due to better risk stratification and tailored therapies, the disease’s unpredictable course and its overlap with normal pregnancy physiology continue to pose diagnostic and therapeutic challenges. This systematic review highlights the critical importance of preconception planning, multidisciplinary care, and close monitoring throughout pregnancy. Only through rigorous, large-scale, and standardized research efforts can we advance precision medicine approaches for women with lupus and achieve optimal, individualized care outcomes.

## Figures and Tables

**Table 1:** Characteristics of the included studies.

Authors	Citation	Country	Type of Study	Number of Pregnancies	Study Population
Alshohaib, 2009	[7]	UAE	PC	20	LN pregnancies
Urowitz et al., 1993	[8]	N/A	CC	N/A	SLE vs. non-SLE pregnancies
Wagner et al., 2009	[9]	USA	RC	N/A	SLE pregnancies
Clowse et al., 2008	[10]	USA	Comparative	16.7 million	SLE vs. non-SLE pregnancies
Ahmed et al., 2020	[11]	Libya	RC	60	SLE pregnancies
Al Arfaj and Khalil, 2010	[12]	UAE	RC	396	Pre- and post-SLE pregnancies
Andrade et al., 2006	[13]	USA	RC	63	SLE pregnancies
Aydin et al., 2014	[14]	Turkey	Observational	N/A	HELPP individuals
Bertolaccini et al., 2007	[15]	England	CC	123	SLE vs. non-SLE pregnancies
Brucato et al., 2002	[16]	Italy	PC	111	Ro+ vs. Ro-SLE pregnancies
Buyon et al., 2015	[17]	USA and Canada	PC	385	SLE pregnancies
Cavallasca et al., 2008	[18]	Argentina	RC	72	SLE pregnancies
Chakravarty et al., 2005	[19]	USA	RC	63	SLE pregnancies
Chen et al., 2015	[20]	China	RC	83	SLE pregnancies
Chandran et al., 2005	[21]	India	RC	52	SLE pregnancies
Clark et al., 2003	[22]	Canada	RC	88	SLE pregnancies
Clowse et al., 2011	[23]	USA	RC	267	SLE pregnancies
Daskalakis et al., 1998	[24]	Greece	RC	12	SLE pregnancies
Rokutanda et al., 2013	[25]	USA	PC	40	SLE pregnancies
Cortes-Hernandez et al., 2002	[26]	Spain	PC	103	SLE pregnancies
Derksen et al., 1994	[27]	Holland	PC	35	SLE pregnancies
Eudy et al., 2018	[28]	USA	RC	398	SLE pregnancies
Gaballa et al., 2012	[29]	Egypt	PC	40	SLE vs. non-SLE pregnancies
Georgiou et al., 2000	[30]	UK	CC	118	SLE vs. non-SLE pregnancies
Giancotti et al., 2011	[31]	Italy	PC	11	SLE pregnancies
Huang et al., 2019	[32]	China	Case Report	1	SLE onset after pregnancy
Lê Thi Huong et al., 2001	[33]	France	PC	116	SLE pregnancies
Lê Thi Huong et al., 1997	[34]	France	PC	62	SLE pregnancies
Imbasciati et al., 2009	[35]	Italy	RC	113	LN pregnancies
Jaeggi et al., 2010	[36]	Canada	CC	186	Anti-Ro-positive pregnancies
Julkunen et al., 1993	[37]	Finland	Retrospective CC	242	SLE vs. non-SLE pregnancies
Julkunen et al., 1993	[38]	Finland	RC	26	SLE pregnancies
Kim and Lee, 2008	[39]	South Korea	CC	105	SLE vs. non-SLE pregnancies
Leaños-Miranda et al., 2007	[40]	Mexico	PC	250	SLE vs. non-SLE pregnancies
Liu et al., 2012	[41]	China	RC	111	SLE pregnancies
Lockshin et al., 1984	[42]	USA	Prospective CC	144	Antiphospholipid pregnancies
Mavragani et al., 1998	[43]	Greece	RC	154	Anti-Ro vs. non-SLE pregnancies
Mintz et al., 1996	[44]	N/A	PC	102	SLE pregnancies
Mokbel et al., 2013	[45]	Egypt	PC	37	SLE pregnancies
Molad et al., 2005	[46]	Israel	PC	29	SLE pregnancies
Moroni et al., 2002	[47]	Italy	RC	70	LN pregnancies
Mosca et al., 2007	[48]	Italy	PC	21	SLE pregnancies
Nossent and Swaak, 1990	[49]	Netherlands	CC	63	SLE vs. non-SLE pregnancies
Ogasawara et al., 1995	[50]	Japan	PC	12	SLE pregnancies
Oviasu et al., 1991	[51]	UK	RC	53	SLE pregnancies
Petri and Allbritton, 1993	[52]	USA	Retrospective CC	1403	SLE vs. non-SLE pregnancies
Rubbert et al., 1992	[53]	USA	Retrospective CC	21	SLE pregnancies vs. SLE individuals
Salazar-Páramo et al., 2002	[54]	Mexico	CC	30	SLE vs. non-SLE pregnancies
Salomonsson et al., 2002	[55]	Sweden	RC	34	Anti-Ro-positive pregnancies
Sittiwangkul et al., 1999	[56]	Thailand	RC	48	SLE pregnancies
Stagnaro-Green et al., 2011	[57]	USA	RC	63	SLE pregnancies
Spence et al., 2006	[58]	Canada	RC	102	SLE pregnancies
Surita et al., 2007	[59]	N/A	PC	N/A	SLE pregnancies
Tandon et al., 2004	[60]	Canada	Prospective CC	156	SLE pregnancies vs. healthy individuals
Tozman et al., 1980	[61]	N/A	RC	24	SLE pregnancies
Whitelaw et al., 2008	[62]	South Africa	RC	47	SLE pregnancies
Wong et al., 1991	[63]	N/A	PC	29	SLE pregnancies
Wong et al., 2006	[64]	Taiwan	RC	24	SLE pregnancies
Yuen et al., 2008	[65]	Canada	CC/Review	242	SLE pregnancies vs. healthy individuals
Zhao et al., 2013	[66]	China	RC	48	New-onset SLE in pregnancy
Yang et al., 2025	[67]	China	SRMA	N/A	SLE pregnancies across multiple global cohorts
Cajamarca-Baron et al., 2025	[68]	Latin America	SRMA	N/A	SLE pregnancies
Zhang et al., 2025	[69]	China	PC (multicenter)	800	SLE pregnancies
Wind et al., 2024	[70]	Netherlands	SRMA	N/A	SLE pregnancies
Dai et al., 2024	[71]	China	PC	300	SLE pregnancies
Zucchi et al., 2023	[72]	Italy, Germany	Review	N/A	SLE pregnancies

SLE = systemic lupus erythematosus, LN = lupus nephritis, RC = retrospective cohort study, PC = prospective cohort study, CC = case–control study, SRMA = systematic review and meta-analysis.

**Table 2:** Evaluation of the quality of the included studies using the Newcastle–Ottawa scale.

	Study	Selection (Max: 4)	Comparability (Max: 2)	Outcome-Exposure (Max: 3)	Total Score (Max: 9)	Comments
Alshohaib, 2009	[7]	4	2	2	8	Excellent methodological rigor with consistent adjustment for key factors
Urowitz et al., 1993	[8]	4	2	2	8	Exposure and outcome criteria clearly optimized
Wagner et al., 2009	[9]	3	2	2	7	Well-structured with minor concerns on follow-up and comparability
Clowse et al., 2008	[10]	3	2	2	7	Limited sample robustness; fair adjustment strategies
Ahmed et al., 2020	[11]	3	1	2	6	Adequate design with limited adjustments for confounding variables
Al Arfaj and Khalil, 2010	[12]	3	1	2	6	Acceptable structure, though lacking multicenter validation
Andrade et al., 2006	[13]	3	2	2	7	Robust framework; minor gaps in outcome follow-up
Aydin et al., 2014	[14]	3	2	2	7	Sound design with thorough comparability measures
Bertolaccini et al., 2007	[15]	3	2	2	7	Limited in sample diversity; exposure ascertainment is solid
Brucato et al., 2002	[16]	3	1	2	6	Lacks full confounder control; appropriate outcome definition
Buyon et al., 2015	[17]	3	1	2	6	Thorough assessment, though external generalizability could be improved
Cavallasca et al., 2008	[18]	4	2	3	9	Strong quality design; exceptional clarity in exposure/outcome tracking
Chakravarty et al., 2005	[19]	3	1	2	6	Confounding adjustment suboptimal; otherwise well-constructed
Chen et al., 2015	[20]	3	1	2	6	Exposure criteria adequate; limited scope in geographic population
Chandran et al., 2005	[21]	3	1	2	6	Simple but effective methodology; lacks longitudinal follow-up
Clark et al., 2003	[22]	3	2	2	7	Methodologically solid; some underreporting in control variables
Clowse et al., 2011	[23]	3	1	2	6	Strong internal validity; external replication needed
Daskalakis et al., 1998	[24]	3	2	2	7	Effective methods; lacks temporal control in exposure assessment
Rokutanda et al., 2013	[25]	4	2	3	9	Strong multicenter analysis with detailed outcome tracking
Cortes-Hernandez et al., 2002	[26]	3	2	2	7	Well-powered cohort; minor outcome documentation concerns
Derksen et al., 1994	[27]	4	2	3	9	Excellent cohort structure; rigorous adjustment practices
Eudy et al., 2018	[28]	4	2	2	8	Multicenter validation strengthens findings; strong confounder control
Gaballa et al., 2012	[29]	3	1	2	6	Strong statistical methodology; selection bias possible
Georgiou et al., 2000	[30]	3	2	2	7	Sample limitations reduce generalizability; otherwise, well-conceived
Giancotti et al., 2011	[31]	4	2	3	9	Very strong methodology, broad representativeness, and proper adjustment for confounders
Huang et al., 2019	[32]	4	2	2	8	Top-tier quality with proper comparator handling
Lê Thi Huong et al., 2001	[33]	4	2	2	8	Well-adjusted for bias; sample selection slightly limited
Lê Thi Huong et al., 1997	[34]	3	2	2	7	Well-structured with minor concerns on follow-up or comparability
Imbasciati et al., 2009	[35]	3	2	2	7	Well-structured with minor concerns on follow-up or comparability
Jaeggi et al., 2010	[36]	4	2	2	8	Careful exposure assessment; modest loss to follow-up
Julkunen et al., 1993	[37]	3	1	2	6	Methodology is clear; limited diversity in the sample
Julkunen et al., 1993	[38]	3	1	2	6	Clear methodology, but comparability is not fully addressed
Kim and Lee, 2008	[39]	4	2	3	9	Very strong methodology, broad representativeness, and proper adjustment for confounders
Leaños-Miranda et al., 2007	[40]	3	1	2	6	Moderate design limitations, single-center, and lacking comparability
Liu et al., 2012	[41]	4	2	2	8	Sound design with thorough comparability measures
Lockshin et al., 1984	[42]	4	2	2	8	Strong quality design; exceptional clarity
Mavragani et al., 1998	[43]	4	2	2	8	Excellent comparability design; generalizability is strong
Mintz et al., 1996	[44]	3	1	2	6	Appropriate structure; limited statistical depth
Mokbel et al., 2013	[45]	4	2	3	9	Transparent methods with robust validation procedures
Molad et al., 2005	[46]	3	1	2	6	Well-structured, minor limitations in selection
Moroni et al., 2002	[47]	4	2	3	9	Very strong methodology, broad representativeness, and proper adjustment for confounders
Mosca et al., 2007	[48]	3	1	2	6	Clear methodology, but comparability is not fully addressed
Nossent and Swaak, 1990	[49]	4	2	2	8	Single-center limits external validity; methods are reliable
Ogasawara et al., 1995	[50]	4	2	2	8	Very strong methodology, broad representativeness, and proper adjustment for confounders
Oviasu et al., 1991	[51]	3	2	2	7	Sufficiently powered but lacking external replication
Petri and Allbritton, 1993	[52]	3	1	2	6	Clear methodology, but comparability is not fully addressed
Rubbert et al., 1992	[53]	3	1	2	6	Modest design with adequate outcome specification
Salazar-Páramo et al., 2002	[54]	3	2	2	7	Sound overall, though it lacked in-depth comparator documentation
Salomonsson et al., 2002	[55]	4	2	2	8	Very strong methodology, broad representativeness, and proper adjustment for confounders
Sittiwangkul et al., 1999	[56]	3	1	2	6	The retrospective nature limits clarity
Stagnaro-Green et al., 2011	[57]	3	2	2	7	Well-structured with minor concerns on follow-up and comparability
Spence et al., 2006	[58]	3	1	2	6	Moderate design limitations, single-center, and lacking comparability
Surita et al., 2007	[59]	4	2	3	9	Very strong methodology, broad representativeness, and proper adjustment for confounders
Tandon et al., 2004	[60]	4	2	2	8	High reliability with well-structured outcome domains
Tozman et al., 1980	[61]	4	2	3	9	Excellent scoring across all NOS domains
Whitelaw et al., 2008	[62]	4	2	2	8	Very strong methodology, broad representativeness, and proper adjustment for confounders
Wong et al., 1991	[63]	4	2	2	8	Excellent methodological rigor with consistent adjustment for key factors
Wong et al., 2006	[64]	3	2	2	7	Sound overall, though it lacked in-depth comparator documentation
Yuen et al., 2008	[65]	3	2	2	7	Moderate design strength with acceptable bias control
Zhao et al., 2013	[66]	3	1	2	6	Reliable outcome documentation and exposure assessment, study groups show some baseline differences that may affect interpretation
Yang et al., 2025	[67]	4	2	2	8	Well-conducted with robust selection criteria and moderate uniformity in outcome measures, minor variability in exposure definitions
Cajamarca-Baron et al., 2025	[68]	4	2	2	8	Comprehensive meta-analysis with diverse sources, comparability criteria well met, but outcomes reported with some heterogeneity between studies
Zhang et al., 2025	[69]	4	2	3	9	High-quality, large-scale, with clear preconception metrics, strong follow-up integrity, detailed outcome classification, and generalizability enhanced by multicenter design
Wind et al., 2024	[70]	4	2	2	8	Well-structured use of inclusion parameters, comparability of predictors was well addressed, though outcome definitions varied slightly across studies
Dai et al., 2024	[71]	4	2	3	9	Strong predictor–outcome alignment and comprehensive adjustment for confounding variables, rigorous lab-based risk factor evaluation supports robustness
Zucchi et al., 2023	[72]	2	1	2	5	Lacks systematic methods or bias mitigation tools, limiting the reliability of its conclusions

**Table 3:** Frequency of reported adverse pregnancy outcomes (APOs) across the 65 included studies.

Adverse Pregnancy Outcome	No. of Studies Reporting APOs (*n =* 65)	% of Studies
Preterm birth	34	52%
Miscarriage	29	45%
Intrauterine growth restriction (IUGR)	17	26%
Stillbirth	11	17%
Low birth weight	10	15%
Neonatal death	7	11%

## Data Availability

No new data were created or analyzed in this study.

## Supplementary Materials

The following supporting information can be downloaded at: https://www.mdpi.com/article/10.3390/medsci13030174/s1, PRISMA 2020 checklist.

## Abbreviations

The following abbreviations are used in this manuscript:

Abbreviation	Full Form
SLE	Systemic lupus erythematosus
LN	Lupus nephritis
APO	Adverse pregnancy outcome
NLS	Neonatal lupus syndrome
aPL	Antiphospholipid antibodies
CHB	Complete heart block
NICU	Neonatal intensive care unit
IUGR	Intrauterine growth restriction
HELLPs	Hemolysis with elevated liver tests and low platelets
HCQ	Hydroxychloroquine
APS	Antiphospholipid syndrome
ANAs	Anti-nuclear antibodies
dsDNA	Double-stranded DNA
Th1	T helper 1 cells
Th2	T helper 2 cells
IL-10	Interleukin-10
IL-6	Interleukin-6
hCG	Human chorionic gonadotropin
PRL	Prolactin
IVIg	Intravenous immunoglobulin
RCTs	Randomized controlled trials
NOS	Newcastle–Ottawa scale
PRISMA	Preferred Reporting Items for Systematic Reviews and Meta-Analyses
PICOS	Population, Intervention, Comparison, Outcome, Study design
RC	Retrospective cohort study
PC	Prospective cohort study
CC	Case–control study
SRMA	Systematic review and meta-analysis
